# T1 mapping in daily CMR practice

**DOI:** 10.1186/1532-429X-16-S1-P232

**Published:** 2014-01-16

**Authors:** Jonathan Nadjiri, Eva Hendrich, Albrecht Will, Cornelia Pankalla, Stefan Martinoff, Martin Hadamitzky

**Affiliations:** 1 Department of Radiology and Nuclear Medicine, Deutsches Herzzentrum München, Munich, Bavaria, Germany

## Background

Dual T1 mapping allows for a comprehensive assessment of myocardial tissue by combining detection of edema in the native scan and quantification of extracellular volume (ECV) after administration of Gadolinium. Recent studies proved the diagnostic value of T1 mapping in different pathologies; the aim of this study was, to evaluate the practicability and robustness of T1 mapping in assessing common pathologies in daily CMR practice.

## Methods

Since October 2012, we investigated 140 patients undergoing clinically indicated CMR examination by perfoming additional T1 mapping sequences which were not used for clinical diagnosis. We used a modified Short Modified Look Locker Inversion Recovery (ShMOLLI) sequence with 3 inversion pulses and a 4-(1)-3-(1)-2 readout pattern. The post Gadolinium scan for extra cellular volume calculation was performed 10 min. after administration of a bolus of 0.2 mmol/kg body weight gadopentetate dimeglumine. Diagnosis was based on clinical information and standard sequences for assessment of myocardial tissue comprising native T2 weighted dark blood turbospin echo (tse) sequences, pre and early post gadolinium T1 weighted tse sequences and inversion recovery spoiled gradient echo sequences for late enhancement. Assessment of T1 relaxation time and ECV values was based on 3 short axis views and 1 long axis view using the AHA/ACC 17-segment model.

## Results

Native T1 relaxation time and ECV for the different pathologies are summarized in Table 1. For all pathologies either native T1 or ECV showed a significant difference when compared with healthy individuals. Particularly high native T1 values were observed in acute myocarditis, hypertrophic cardiomyopathy and amyoloidosis, a high ECV was found in myocardial infarction, sarcoidosis and amyoloidosis. Image quality was sometimes limited by incomplete motion correction, particularly in the long axis views and by partial volume effects in the apical short axis views.

**Table 1:** T1 relaxing times and ECV in patients collective

	T1 relaxing time ± SD	p-value	ECV ± SD	p-value
Normal result:	906 ms ± 36 ms	reference	25,5% ± 2,7%	reference

DCM:	975 ms ± 71 ms	p < 0,001	30,1% ± 4,5%	p < 0,001

HCM:	980 ms ± 101 ms	p < 0,001	31,4% ± 6,2%	p < 0,001

Acute myocardial infarction:	973 ms ± 61 ms	p < 0,001	39,9% ± 8,6%	p < 0,001

Chronic myocardial infarction:	934 ms ± 131 ms	p = 0,18	39,4% ± 11,9%	p < 0,001

Acute myocarditis:	1017 ms ± 59 ms	p < 0,001	38,2% ± 8,9%	p < 0,001

Chronic myocarditis:	955 ms ± 45 ms	p < 0,001	27,9% ± 5,1%	p = 0,028

Sarcoidosis:	946 ms ± 66 ms	p < 0,001	35,1% ± 9,0%	p < 0,001

Amyloidosis:	1078 ms ± 54 ms	p < 0,001	58,1% ± 10,4%	p < 0,001

## Conclusions

T1 mapping and ECV correlated well with myocardial alterations in commonly diagnosed cardiac disorders. It proved reliable and robust in daily clinical practice and allows for a good differentiation between normal findings and pcommon pathological CMR diagnoses. The combined use of native T1 and ECV quantification is promising approach for comprehensive assessment of the myocardium and may improve diagnostic accuracy of CMR in myocardial disease.

## Funding

None.

